# Association of early enoxaparin prophylactic anticoagulation with ICU mortality in critically ill patients with chronic obstructive pulmonary disease: a machine learning-based retrospective cohort study

**DOI:** 10.3389/fphar.2025.1588846

**Published:** 2025-05-12

**Authors:** Shan Lin, Jing Zhang, Xin Dang, Qingyuan Zhan

**Affiliations:** ^1^ Department of Respiratory and Critical Care Medicine, Affiliated Hospital of North Sichuan Medical College, Nanchong, Sichuan, China; ^2^ Department of Otolaryngology Head and Neck Surgery, Affiliated Hospital of North Sichuan Medical College, Nanchong, Sichuan, China; ^3^ Department of Pulmonary and Critical Care Medicine, Center of Respiratory Medicine, National Center for Respiratory Medicine, China-Japan Friendship Hospital, Beijing, China

**Keywords:** enoxaparin, prophylactic anticoagulation, chronic obstructive pulmonary disease, critical care, prognosis, machine learning

## Abstract

**Background:**

Chronic obstructive pulmonary disease (COPD) is a major contributor to global morbidity and mortality, particularly during acute exacerbations that frequently require intensive care unit (ICU) admissions. Considering the hypercoagulability associated with COPD, which intensifies during acute episodes, prophylactic anticoagulation therapy may help reduce ICU mortality. However, this potential has not been explored specifically in this population of patients.

**Methods:**

We conducted a retrospective cohort study using data from the Medical Information Mart for Intensive Care IV, spanning patient records from 2008 to 2019 at the Beth Israel Deaconess Medical Center in Boston. This study focused on critically ill patients with COPD, employing feature selection methods, to identify key variables influencing clinical outcomes. The impact of prophylactic enoxaparin on prognosis was assessed using logistic regression models and Kaplan–Meier survival analysis.

**Results:**

Our analysis included 4,433 critically ill patients with COPD, of whom 446 received enoxaparin within the first 72 h of ICU admission. The primary analysis showed that patients treated with enoxaparin experienced a 48% lower ICU mortality (odds ratio 0.52 [95% confidence interval 0.31–0.86]) than that of those not treated with enoxaparin, with an E-value of 3.26. This association between enoxaparin use and lower ICU mortality persisted across all subgroups examined. Additionally, a visual analysis of patients with varying Oxford acute severity of illness score (OASIS) indicated that early enoxaparin use was linked to an improved prognosis in critically ill patients with COPD who had higher OASIS than in those without.

**Conclusion:**

Early initiation of prophylactic enoxaparin therapy was significantly associated with low ICU mortality in critically ill patients with COPD, especially in high-risk subgroups. These findings support the need for randomized controlled trials to confirm the effectiveness of thromboprophylaxis in this specific patient population and to evaluate the potential bleeding risks.

## Introduction

Chronic obstructive pulmonary disease (COPD) is a widespread chronic respiratory condition that significantly affects global health and impacts approximately 10% of the global population. It is one of the leading causes of death and disability among chronic diseases, as reported in the Global Burden of Disease study (GBD Chronic Respiratory Disease Collaborators, 2017; GBD Disease and Injury Incidence and Prevalence Collaborators, 2015; Wang et al., 2018). Acute exacerbations of COPD (AECOPD) frequently result in complications, such as respiratory failure, severe infections, and heart failure, often necessitating intensive care unit (ICU) admissions. Studies indicate that 10%–20% of patients with AECOPD require intensive care, a figure that increases to 20%–30% in cases accompanied by severe hypoxemia, hypercapnia, acidosis, or impaired consciousness (Esteban et al., 2002; Seneff et al., 1995). The need for ICU care increases sharply with disease severity, the presence of comorbidities, and the complexity of complications. Unfortunately, once admitted to the ICU, patients with AECOPD face a high risk of mortality, with rates ranging from 10% to 30% (Alzaabi et al., 2024; Xie et al., 2024).

COPD, particularly during the moderate-to-severe stages and acute exacerbations, is characterized by a persistent systemic inflammatory state. Chronic inflammation significantly contributes to endothelial dysfunction, platelet activation, and hypercoagulability, thereby increasing the risk of thrombotic events (Agapakis et al., 2016; Fawzy et al., 2023). Further compounding these risks are comorbidities, such as pulmonary hypertension and right ventricular dysfunction, which are commonly seen in the advanced stages of COPD. These conditions alter blood rheology and promote venous stasis (Cavaillès et al., 2013). Clinical studies have consistently shown a high prevalence of coagulation abnormalities—including elevated D-dimer levels, increased coagulation factor activity, and enhanced platelet aggregation—in patients with COPD. Notably, most of patients with COPD exhibit measurable disturbances in their coagulation–fibrinolysis balance, particularly during acute exacerbations (Duvoix et al., 2013; Kyriakopoulos et al., 2021). Hospitalized patients with AECOPD also exhibit significantly higher D-dimer levels than those of patients in stable condition, suggesting an elevated thrombotic risk (Hu et al., 2016). The degree of coagulation dysfunction correlates with disease progression and is linked to poor clinical outcomes, including prolonged hospital stay and increased mortality rates (Husebø et al., 2021).

In critically ill patients with COPD requiring ICU admission, factors, such as immobilization, mechanical ventilation, and the use of sedatives, further exacerbate their already heightened hypercoagulable state, significantly increasing the risk of venous thromboembolism (VTE) (Kahn et al., 2012). Although current guidelines recommend prophylactic anticoagulation to mitigate the risk of VTE in critically ill populations (Barbar et al., 2010), evidence specific to patients with COPD remains sparse. Low-molecular-weight heparins (LMWHs), such as enoxaparin, are commonly used in the ICU to prevent VTE. However, the effect of LMWHs on mortality rates among patients with COPD remains uncertain (Samama et al., 1999). Given the unique pathophysiology of COPD, which includes chronic inflammation and endothelial dysfunction, LMWHs may offer additional benefits, such as anti-inflammatory and endothelial-protective effects (Beitland et al., 2015). This highlights the urgent need for targeted research to evaluate the mortality benefits and risk-benefit ratio of prophylactic anticoagulation in this high-risk, pathophysiologically distinct group.

## Methods

### Data sources

This study used data from the Medical Information Mart for Intensive Care IV, a comprehensive database that encapsulates electronic health records of patients admitted to the Beth Israel Deaconess Medical Center in Boston, Massachusetts, United States, between 2008 and 2019 (Johnson AEW. et al., 2023; Johnson A. et al., 2023). Access to the database was authorized by the Institutional Review Boards of the Beth Israel Deaconess Medical Center and Massachusetts Institute of Technology in Cambridge, Massachusetts, United States (record ID:49780033). Patient consent was waived as the data were anonymized, ensuring privacy and adherence to ethical standards.

### Study population

This study targeted patients aged 18 years or older who were admitted to the ICU with COPD. We implemented a multistage screening process to establish the cohort, adhering to the following inclusion criteria: 1) a confirmed COPD diagnosis characterized by persistent airflow limitation, verified by a post-bronchodilator FEV1/FVC ratio below 0.70; 2) the patient’s first ICU admission; and 3) an ICU length of stay of at least 24 h. To focus on the impact of prophylactic anticoagulation with enoxaparin, initiated within 72 h of ICU admission, on ICU mortality and to minimize confounding from therapeutic anticoagulation, we excluded patients with comorbid atrial fibrillation or a history of lower-extremity thrombosis. Laboratory indicators were the first results obtained within 24 h of ICU admission. The data extraction methods refer to our previous studies (Lin et al., 2023; Lin and Lin, 2024; Lin et al., 2021).

Data collection included various parameters: demographic characteristics (age and sex), disease severity markers (Oxford acute severity of illness [OASIS] score), comorbidity burden (Charlson Comorbidity Index), organ support interventions (mechanical ventilation and continuous renal replacement therapy [CRRT]), and infection-related parameters (sepsis status and laboratory biomarkers). For patients with multiple ICU admissions, only data from the initial admission were analyzed to maintain data independence.

## Outcomes

The primary endpoint of the study was the effect of 72 h of prophylactic anticoagulation with enoxaparin on ICU mortality in patients with COPD admitted to the ICU, and the secondary endpoint was 28-day mortality and length of ICU stay.

### Covariate filtering methods

**FIGURE 1:**
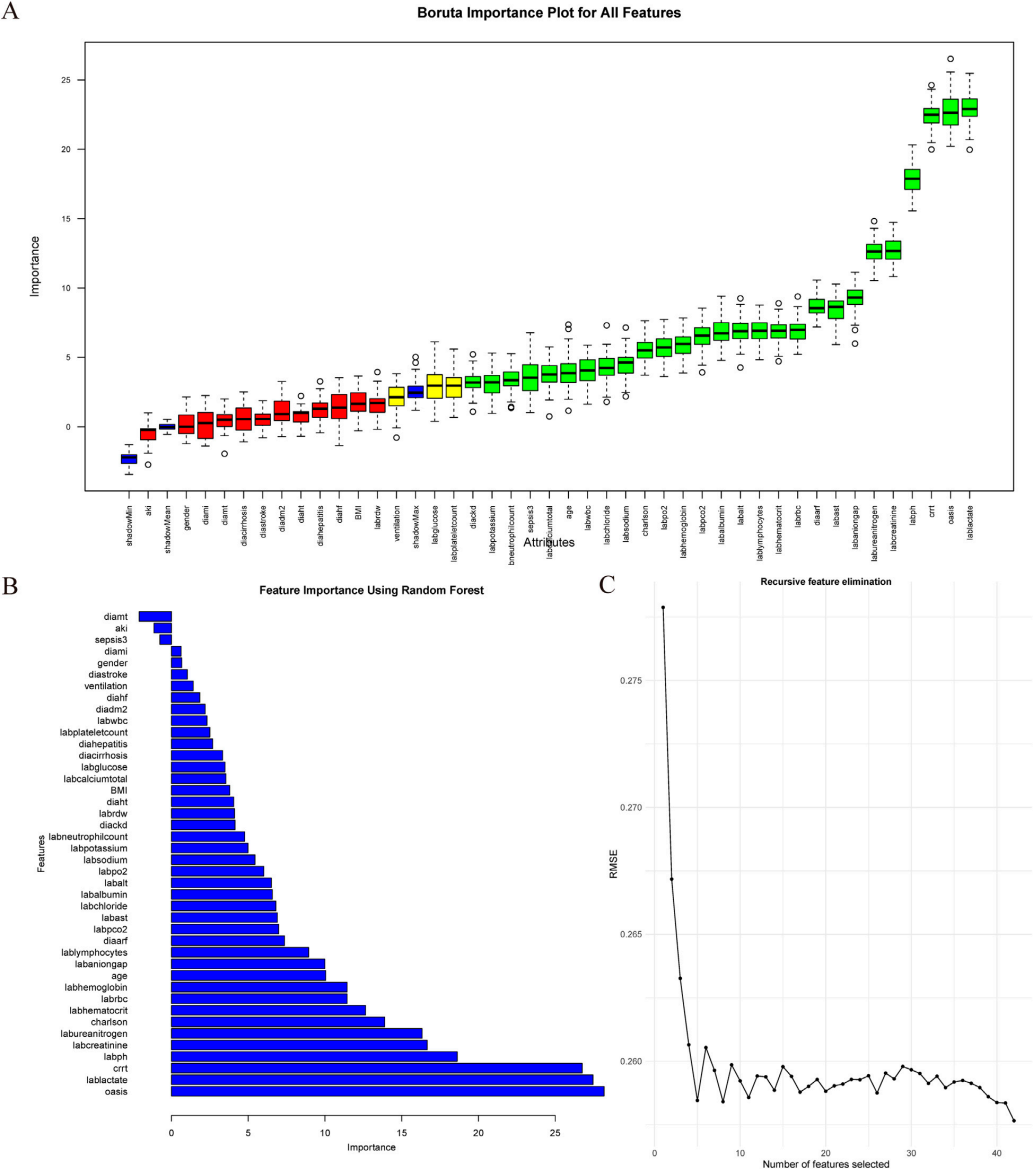
Feature variable screening. **(A)** Feature variable screening using Boruta; **(B)** Feature variable screening using RF; **(C)** Feature variable screening using RFE. Abbreviations: RF: random forest; RFE: recursive feature elimination.

In this study, we applied three distinct feature selection methodologies—Recursive Feature Elimination, Random Forest Importance Scoring, and the Boruta Algorithm—to systematically identify variables strongly associated with clinical outcomes. Recursive Feature Elimination, a wrapper-based approach, employs an iterative process to eliminate the least informative features by training predictive models (including support vector machines or random forests) and evaluating the feature contributions through cross-validation (Halder et al., 2025; Roy, 2025). This method demonstrates high efficacy in reducing feature redundancy, particularly for small-scale datasets. Random Forest Importance Scoring quantifies the predictive relevance of variables using two primary metrics: “IncNodePurity” (measuring impurity reduction at decision tree nodes) and “mean decrease in accuracy” (assessing the decline in model performance when feature values are permuted) (Rohan et al., 2025). This technique excels at capturing nonlinear relations and managing high-dimensional data structures. The Boruta Algorithm, a robust wrapper method, enhances feature selection reliability by statistically comparing original features against artificially generated “shadow features” (randomized permutations of original data) through Z-score testing, thereby effectively controlling false-positive rates (Wu et al., 2025). To ensure robustness and mitigate potential biases inherent in the individual methods, we integrated the results of all three approaches. The consensus among these methods identified five critical variables: OASIS, CRRT, lactate, pH, and creatinine, which were prioritized based on their consistent statistical significance and biological plausibility in influencing clinical outcomes (Figure 1).

### Statistical analysis

Data for continuous variables are presented as either mean ± standard deviation or as median with interquartile range, depending on the distribution. Group characteristics were analyzed using Student’s t-test for normally distributed variables and the Wilcoxon rank-sum test for non-normally distributed variables. Categorical variables were reported as counts and percentages and compared using the chi-square test. Following a machine learning-based variable screening process, five key variables were identified for inclusion in the final regression model: OASIS score, CRRT, lactate levels, pH, and creatinine levels.

We examined the effect of the prophylactic use of enoxaparin on ICU mortality among patients with COPD admitted to the ICU using three logistic regression models: Model 1 was unadjusted, Model 2 was adjusted for common clinical variables, such as age and sex, and Model 3 incorporated variables identified by machine learning. To assess the potential impact of unaccounted confounders on the observed association between prophylactic enoxaparin use and ICU mortality, we calculated the E-value (VanderWeele and Ding, 2017; Haneuse et al., 2019). The E-value is a sensitivity analysis metric used to assess the effect of unmeasured confounders on caUnited Statesl effect estimates in observational studies. It quantifies the minimum strength of association that unmeasured confounders need to have with both the exposure (treatment) and outcome to fully explain the observed exposure–outcome association, controlling for measured covariates. Specifically, a larger E-value indicates that a stronger unmeasured confounding effect is required to refute the study’s findings, whereas a smaller E-value implies that a weaker confounding effect can explain the results. Additionally, we constructed propensity score models to assess the robustness of the results, including covariate-adjusted propensity scores, propensity score matching, and propensity score inverse probability weighting.

Survival differences between groups were illustrated using the Kaplan–Meier method, with significance assessed using time-series tests. Stratified analyses and interaction tests were conducted to examine the consistency of the associations across various subgroups, including demographic characteristics, therapeutic interventions, sepsis, acute kidney injury (AKI), and varying OASIS scores. Data analysis was performed using R software (version 4.4.2), and a p-value of less than 0.05 was considered statistically significant.

## Results

### Basic characteristics of the study population

In this study, we analyzed a cohort of 4,433 critically ill patients with COPD. Within the first 72 h of ICU admission, 10.1% of the patients (446 individuals) received prophylactic anticoagulation with enoxaparin, as detailed in Table 1. Our analysis revealed that 49.13% of the cohort (2,178 patients) had sepsis, and 71.53% (3,171 patients) developed AKI after admission. The incidences of these comorbidities were similar between the groups treated with and without enoxaparin. Notably, the use of CRRT was significantly higher in the enoxaparin group, whereas the use of mechanical ventilation was less frequent than in the group without enoxaparin. Additional clinical characteristics and intergroup comparisons are presented in Table 1.

**TABLE 1:** Characteristics of critically ill patients with COPD.

Variables	All patients (N = 4,433)	Without enoxaparin (N = 3,987)	With enoxaparin (N = 446)	P-value
Age	71.59 ± 11.25	71.72 ± 11.26	70.45 ± 11.10	0.023
Sex				0.066
Male	2310 (52.11%)	2096 (52.57%)	214 (47.98%)	
Female	2123 (47.89%)	1891 (47.43%)	232 (52.02%)	
BMI	29.37 ± 9.10	29.40 ± 9.07	29.04 ± 9.39	0.420
OASIS	31.78 ± 8.51	31.82 ± 8.58	31.46 ± 7.82	0.397
ICU mortality	407 (9.18%)	389 (9.76%)	18 (4.04%)	<0.001
28-day mortality	782 (17.64%)	722 (18.11%)	60 (13.45%)	0.014
Length of ICU stay	2.20 (1.21–4.65)	2.18 (1.20–4.55)	2.56 (1.28–5.05)	0.117
pH	7.35 (7.33–7.38)	7.35 (7.33–7.38)	7.35 (7.34–7.39)	0.178
PaCO_2_	46.76 (41.77–47.73)	46.76 (41.72–47.33)	46.76 (43.00–51.00)	<0.001
PaO_2_	115.01 (69.20–117.67)	115.01 (71.81–122.62)	100.75 (56.62–115.01)	<0.001
Lactate (mmol/L)	2.14 (1.45–2.14)	2.14 (1.45–2.14)	2.14 (1.40–2.14)	0.155
White blood cells (K/uL)	11.50 (8.40–14.95)	11.50 (8.40–14.96)	11.28 (7.82–14.74)	0.102
Red blood cells (m/uL)	3.55 (3.07–4.04)	3.54 (3.07–4.02)	3.59 (3.11–4.18)	0.064
Neutrophil (K/uL)	10.94 (10.94–10.94)	10.94 (10.94–10.94)	10.94 (10.94–10.94)	0.264
Lymphocyte (K/uL)	1.74 (1.61–1.74)	1.74 (1.67–1.74)	1.74 (1.20–1.74)	<0.001
Platelet (K/uL)	193.00 (143.00–249.50)	192.00 (142.00–246.42)	206.00 (149.50–271.50)	<0.001
Hemoglobin (g/dL)	10.50 (9.05–11.90)	10.50 (9.03–11.88)	10.59 (9.10–12.30)	0.282
RDW (%)	14.80 (13.65–16.22)	14.80 (13.65–16.20)	14.82 (13.79–16.30)	0.334
Hematocrit (%)	32.60 (28.37–36.87)	32.52 (28.35–36.75)	32.93 (28.61–38.60)	0.042
Albumin (g/dL)	3.14 (3.14–3.14)	3.14 (3.14–3.14)	3.14 (3.14–3.14)	0.167
Sodium (mEq/L)	138.33 (136.00–141.00)	138.33 (136.00–141.00)	138.00 (135.00–140.50)	0.003
Potassium (mEq/L)	4.26 (3.90–4.65)	4.27 (3.90–4.66)	4.20 (3.90–4.63)	0.145
Calcium (mg/dL)	8.45 (8.03–8.85)	8.45 (8.03–8.85)	8.50 (8.05–8.90)	0.219
Chloride (mEq/L)	102.67 (99.00–106.00)	103.00 (99.00–106.00)	102.00 (97.67–104.65)	<0.001
Glucose (mg/dL)	130.00 (108.00–161.00)	130.00 (108.00–160.33)	132.42 (108.00–166.92)	0.338
Anion gap (mEq/L)	13.50 (11.25–16.00)	13.67 (11.33–16.00)	12.75 (11.00–15.00)	<0.001
Bilirubin (mg/dL)	1.28 (0.60–1.28)	1.28 (0.60–1.28)	1.16 (0.40–1.28)	<0.001
ALT (IU/L)	129.83 (22.00–129.83)	129.83 (22.84–129.83)	77.50 (19.00–129.83)	0.001
AST (IU/L)	210.04 (32.00–210.04)	210.04 (34.00–210.04)	84.34 (25.00–210.04)	<0.001
Creatinine (mg/dL)	1.00 (0.73–1.47)	1.00 (0.75–1.50)	0.90 (0.65–1.17)	<0.001
Ureanitrogen (mg/dL)	20.75 (14.00–32.00)	21.00 (14.00–33.12)	18.73 (13.50–27.29)	<0.001
Sepsis	2178 (49.13%)	1974 (49.51%)	204 (45.74%)	0.131
AKI	3171 (71.53%)	2852 (71.53%)	319 (71.52%)	0.997
AKI Stage				0.169
1	837 (18.88%)	763 (19.14%)	74 (16.59%)	
2	1531 (34.54%)	1358 (34.06%)	173 (38.79%)	
3	803 (18.11%)	731 (18.33%)	72 (16.14%)	
CRRT	192 (4.33%)	185 (4.64%)	7 (1.57%)	0.003
MV	3634 (81.98%)	3247 (81.44%)	387 (86.77%)	0.005
Charlson score	6.71 ± 2.73	6.72 ± 2.71	6.61 ± 2.93	0.412
Comorbidities				
Hypertension	1734 (39.12%)	1555 (39.00%)	179 (40.13%)	0.642
Type II diabetes	1472 (33.21%)	1354 (33.96%)	118 (26.46%)	0.001
Heart failure	1831 (41.30%)	1663 (41.71%)	168 (37.67%)	0.100
Myocardial infarction	596 (13.44%)	556 (13.95%)	40 (8.97%)	0.003
Malignant tumor	845 (19.06%)	748 (18.76%)	97 (21.75%)	0.128
CKD	1068 (24.09%)	1004 (25.18%)	64 (14.35%)	<0.001
Hepatitis	196 (4.42%)	174 (4.36%)	22 (4.93%)	0.580
Cirrhosis	271 (6.11%)	249 (6.25%)	22 (4.93%)	0.273
Stroke	411 (9.27%)	380 (9.53%)	31 (6.95%)	0.075

Abbreviations: OASIS, oxford acute severity of illness score; AKI, acute kidney injury; MV, mechanical ventilation; CRRT, continuous renal replacement therapy; CKD, chronic kidney disease; ICU, intensive care unit; ALT, alanine aminotransferase; AST, aspartate aminotransferase; BMI, body mass index; COPD, chronic obstructive pulmonary disease.

## Clinical outcomes in critically ill patients with COPD

**FIGURE 2:**
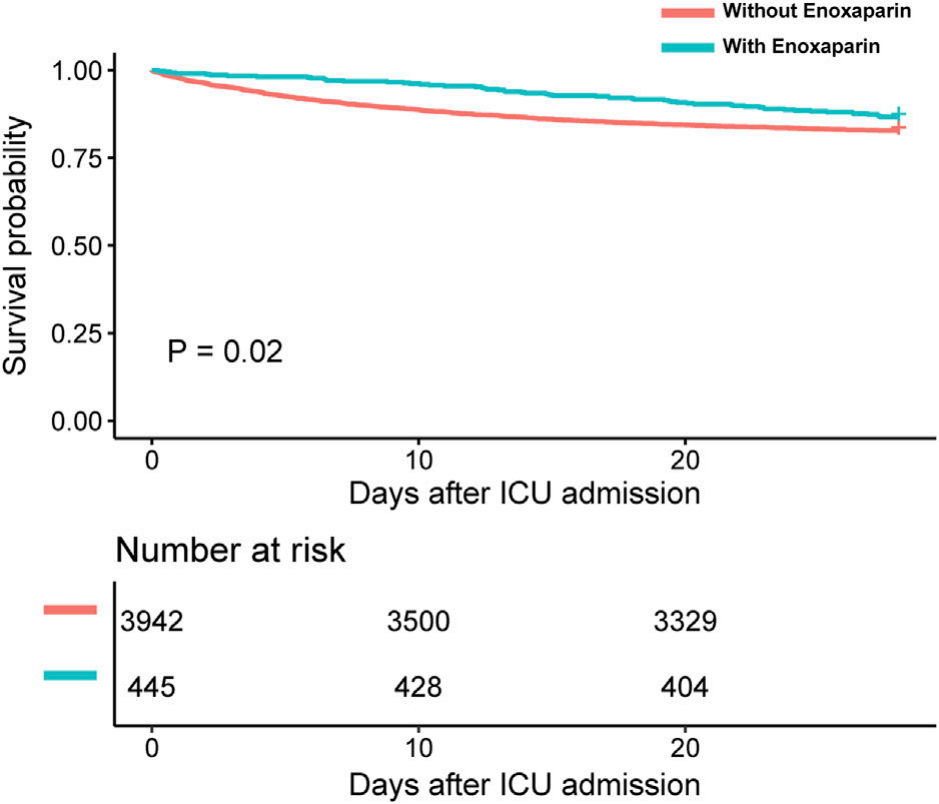
Kaplan-Meier survival curve Abbreviations: ICU: intensive care unit.

The clinical outcomes of this study indicated significantly lower ICU and 28-d mortality rates in the enoxaparin group compared with those in the non-enoxaparin group, with both showing P-values <0.05, as detailed in Table 2. The Kaplan–Meier survival curves further demonstrated a significant survival benefit at 28 days for patients receiving enoxaparin, confirmed by a P-value <0.05, as illustrated in Figure 2. However, there was no significant difference in the length of ICU stay between the groups (p = 0.117).

**TABLE 2:** Clinical outcomes in critically ill patients with COPD.

Clinical outcomes	Without enoxaparin (N = 3,987)	With enoxaparin (N = 446)	*P*-value
ICU mortality, n (%)	389 (9.76%)	18 (4.04%)	<0.001
28-day mortality, n (%)	722 (18.11%)	60 (13.45%)	0.014
Length of ICU stay (days)	2.18 (1.20–4.55)	2.56 (1.28–5.05)	0.117

Abbreviations: COPD, chronic obstructive pulmonary disease; ICU, intensive care unit.

### Associations between enoxaparin use and clinical outcomes

As outlined in Table 3, ICU mortality was consistently lower in the enoxaparin group across all analyzed models. Notably, in Model III, the use of enoxaparin was associated with a 48% decrease in ICU mortality compared with that in patients who did not receive enoxaparin (odds ratio 0.52 [95% confidence interval [CI] 0.31–0.86]). The estimated E-value of 3.26 (with an upper confidence limit of 2.31) indicates that any unmeasured confounders would need to have a relative risk of at least 3.26 in relation to both enoxaparin use and ICU mortality to negate the observed association.

**TABLE 3:** Associations between enoxaparin use and ICU mortality.

Clinical outcome			
ICU mortality	Groups	OR (95% CI)	*P*-value
Crude analysis (Model I)	Without enoxaparin With enoxaparin	Ref. 0.39 (0.24–0.63)	- 0.0001
Multivariable analysis (Model II)	Without enoxaparin With enoxaparin	Ref. 0.47 (0.28–0.79)	- 0.0041
Multivariable analysis based on machine learning (Model III)	Without enoxaparin With enoxaparin	Ref. 0.52 (0.31–0.86)	- 0.0106
Propensity-score models (Model IV)			
Adjusted for propensity score	Without enoxaparin With enoxaparin	Ref. 0.55 (0.33–0.89)	- 0.0158
With matching	Without enoxaparin With enoxaparin	Ref. 0.37 (0.21–0.66)	- 0.0006
With inverse probability weighting	Without enoxaparin With enoxaparin	Ref. 0.40 (0.22–0.70)	- 0.0015

Multivariable analysis (Model II) adjusted for age, sex, BMI, OASIS, charlson, AKI, CRRT, MV, and sepsis.

Multivariable analysis based on machine learning (Model III) adjusted for OASIS, CRRT, lactate, pH, and creatinine.

Propensity score was calculated by OASIS, CRRT, lactate, pH, and creatinine.

Propensity score matching was performed with the use of a 1:1 matching protocol without replacement (greedy-matching algorithm), with a caliper width equal to 0.01 of the standard deviation of the logit of the propensity score.

Inverse probability weighting was used with the same covariates according to the propensity score.

Abbreviations: OR, odds ratio; CI, confidence interval; OASIS, oxford acute severity of illness score; AKI, acute kidney injury; MV, mechanical ventilation; CRRT, continuous renal replacement therapy.

**TABLE 4:** Effect size of enoxaparin use on ICU mortality in prespecified and exploratory subgroups in each subgroup.

Y = ICU mortality	Adjusted model			
	Without enoxaparin	With enoxaparin, OR (95% CI)	*P*-value	*P* for interaction
Age (years)				0.6315
<65	1.0	0.42 (0.13–1.39)	0.1567	
≥65	1.0	0.56 (0.32–0.97)	0.0384	
Sex				0.7637
Male	1.0	0.61 (0.31–1.21)	0.1555	
Female	1.0	0.46 (0.22–0.96)	0.0385	
OASIS				0.4951
<30	1.0	0.26 (0.06–1.17)	0.0788	
≥30	1.0	0.55 (0.32–0.95)	0.0305	
pH value				0.1526
<7.35	1.0	0.72 (0.38–1.38)	0.3243	
≥7.35	1.0	0.36 (0.15–0.82)	0.0151	
Lactate (mmol/L)				0.3269
<2	1.0	0.66 (0.32–1.36)	0.2602	
≥2	1.0	0.41 (0.21–0.84)	0.0139	
Creatinine (mg/dL)				0.7189
<1.5	1.0	0.58 (0.33–1.04)	0.0662	
≥1.5	1.0	0.45 (0.15–1.34)	0.1522	
Sepsis				0.4071
No	1.0	0.32 (0.08–1.38)	0.1266	
Yes	1.0	0.55 (0.32–0.95)	0.0332	
AKI				0.5304
No	1.0	0.87 (0.19–3.91)	0.8522	
Yes	1.0	0.48 (0.28–0.82)	0.0073	
MV				0.7637
No	1.0	0.45 (0.06–3.53)	0.4468	
Yes	1.0	0.52 (0.31–0.87)	0.0133	
CRRT				0.7855
No	1.0	0.53 (0.31–0.90)	0.0186	
Yes	1.0	0.39 (0.07–2.23)	0.2925	

Adjusted by OASIS, CRRT, lactate, pH, and creatinine except for the subgroup variable.

Abbreviations: OR, odds ratio; CI, confidence interval; OASIS, oxford acute severity of illness score; AKI, acute kidney injury; MV, mechanical ventilation; CRRT, continuous renal replacement therapy.

### Stratified analyses and interaction tests

**FIGURE 3:**
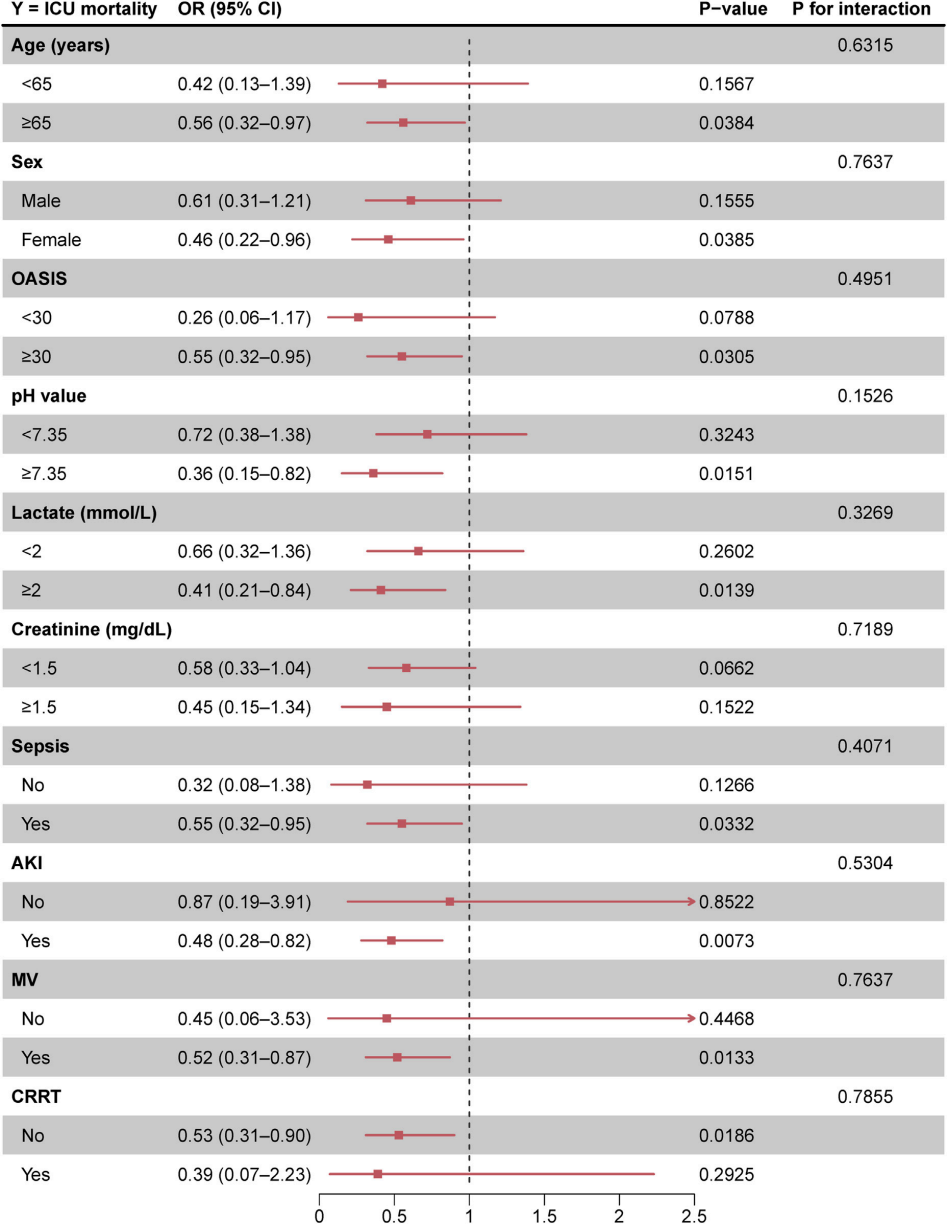
Forest plot of enoxaparin use on ICU mortality in prespecified and exploratory subgroups in each subgroup Abbreviations: OR, odds ratio; CI, confidence interval; OASIS, Oxford acute severity of illness score; AKI, acute kidney injury; MV, mechanical ventilation; CRRT, continuous renal replacement therapy.

**FIGURE 4:**
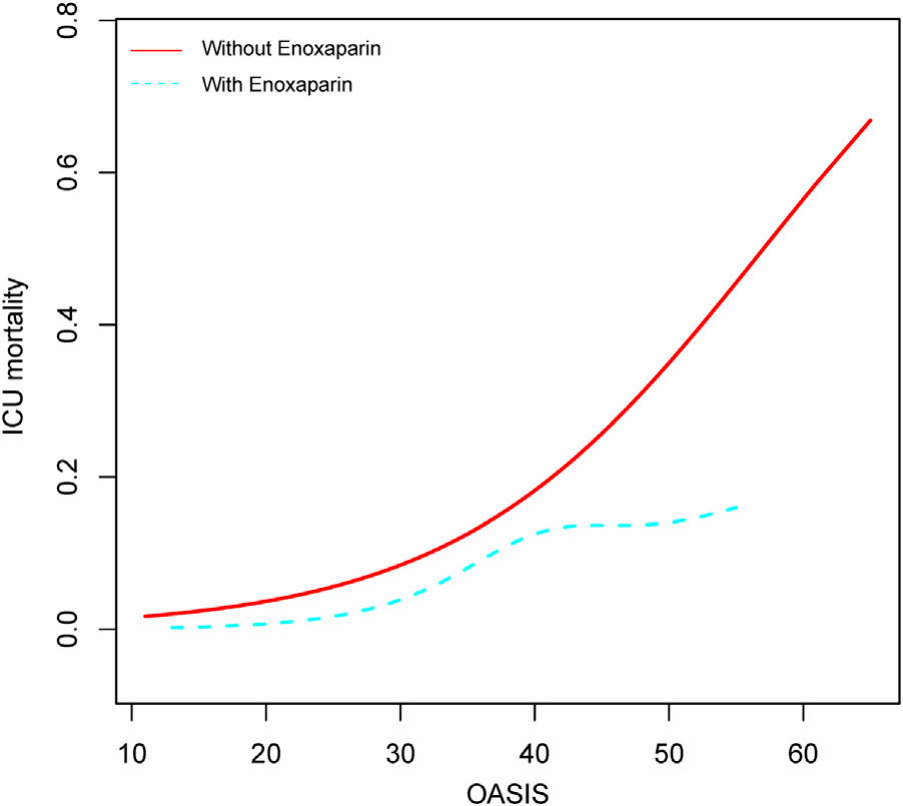
Relationship between OASIS scores and ICU mortality in critically ill patients with COPD Abbreviations: OR, odds ratio; CI, confidence interval; OASIS, Oxford acute severity of illness score.

The association between enoxaparin use and decreased ICU mortality was consistent across all subgroups examined (Table 4 and Figure 3). Specifically, enoxaparin use was linked to lower ICU mortality in patients with severe COPD and an OASIS score of 30 or higher; this association was also observed in patients with a lactate level of 2 mmol/L or greater. Similar benefits were observed in patients with severe COPD complicated by comorbid sepsis, AKI, CRRT, or mechanical ventilation. These findings underscore the robustness of the effect of enoxaparin across various clinical scenarios in the ICU setting. Moreover, as shown in Figure 4, in critically ill patients with COPD and higher OASIS scores, the early use of enoxaparin was associated with a better prognosis than that observed in patients not receiving enoxaparin.

## Discussion

In this study, we analyzed data from a large single-center retrospective cohort of critically ill patients with COPD from the Comprehensive Critical Care Medicine Database to assess the clinical outcomes of enoxaparin use within 72 h of ICU admission and ICU mortality. Our findings indicate that prophylactic anticoagulation with enoxaparin is significantly associated with low ICU mortality.

Critically ill patients with COPD who received prophylactic enoxaparin within 72 h of ICU admission had significantly low ICU mortality (HR = 0.52, 95% CI 0.31–0.86). This aligns with trends indicating a survival benefit from early anticoagulation interventions in various critically ill patient populations, suggesting a potential mechanism for the prevalence of anticoagulation in critical care management. For example, early anticoagulation therapy reduces thrombotic complications and mortality in patients with COVID-19-related ARDS. A multicenter observational study (n = 4,297) demonstrated a 27% reduction in 30-d mortality for patients with neocoronary pneumonia who received prophylactic anticoagulation compared with those who did not (HR = 0.73, 95% CI 0.66–0.81) (Rentsch et al., 2021). Similarly, in patients with sepsis, the early use of heparin was linked to a 22%–30% reduction in risk-adjusted morbidity and mortality (Zou et al., 2022). While the hypercoagulable state in patients with COVID-19 is primarily driven by direct viral damage to endothelial cells, and in patients with COPD by chronic hypoxia and inflammation-mediated coagulation activation, the survival benefits of anticoagulation therapy appear similar (Ackermann et al., 2020; Varga et al., 2020; Petris et al., 2021; Huang et al., 2021). Meta-analyses further support the protective role of early anticoagulation in systemic inflammatory responses, showing significant reductions in 28-d mortality without substantially increasing bleeding risk (Wang et al., 2014; Fan et al., 2016). Moreover, retrospective cohort studies in trauma and surgical ICU patients have shown that prophylactic enoxaparin significantly reduces the incidence of VTE and is strongly correlated with decreased ICU mortality (Andersen et al., 2021; Al Harbi et al., 2013). The risk of VTE in patients with severe COPD is often underestimated, and high D-dimer levels are linked to increased all-cause mortality (Kamstrup et al., 2023). Enoxaparin may indirectly improve prognosis by reducing the risk of VTE.

Concerning the therapeutic doses, notable variability exists in the effectiveness of therapeutic anticoagulation among patients with COVID-19. The HEP-COVID trial demonstrated that therapeutic doses (1 mg/kg or 0.5 mg/kg twice daily) were effective only in non-ICU patients, whereas ICU patients did not see a benefit (Spyropoulos et al., 2021). Additionally, the INSPIRATION trial confirmed that there were no significant differences in mortality or thrombotic events between moderate-dose (1 mg/kg daily) anticoagulation and prophylactic-dose (40 mg daily) anticoagulation in ICU patients. Moreover, bleeding risk was high in the moderate-dose group (Investigators et al., 2021). These findings suggest that prophylactic doses may be adequate to suppress hypercoagulability in critically ill patients with systemic inflammation and that the potential benefits of high doses may be outweighed by an increased risk of bleeding (Mennuni et al., 2021; Mangiafico et al., 2022).

However, the clinical significance of anticoagulation therapy must be assessed in terms of population specificity. In patients with COPD, hypercoagulability is primarily driven by chronic hypoxia and inflammatory factors (including IL-6 and TNF-α). Chronic hypoxia-induced erythrocytosis and secondary platelet activation may exacerbate hypercoagulability, making anticoagulant therapy critical for ameliorating microcirculatory disturbances. The survival benefit of enoxaparin observed in this study may also be related to a reduced risk of VTE. VTE is significantly associated with mortality in ICU patients, and enoxaparin may reduce the incidence of VTE (Quarmby et al., 2024; Veeranki et al., 2021). Additionally, elevated D-dimer levels have been linked to all-cause mortality in patients with COPD, and enoxaparin may improve prognosis by modulating coagulation–inflammation interactions (Hu et al., 2016; Zhang et al., 2022). Although D-dimer-guided dose adjustments in the COVID-19 study were not effective, the need for individualized anticoagulation strategies in patients with COPD remains underexplored (Kyriakopoulos et al., 2021).

This study has several limitations that merit acknowledgment. First, although we employed machine learning techniques to identify and adjust for key confounding variables, the retrospective single-center design may still harbor residual bias from unmeasured factors, such as dynamic fluctuations in inflammatory biomarkers (including D-dimer and fibrinogen) or variations in concurrent therapies (including glucocorticoids and antiplatelet agents), that could influence both enoxaparin use and clinical outcomes. However, sensitivity analyses using E-values indicated that an unmeasured confounder would require a risk ratio of at least 3.26 to fully negate the observed effect, suggesting a relatively low likelihood of significant hidden bias. In addition, we strengthened the robustness of our results by constructing a propensity score model. Second, the study did not assess the impact of enoxaparin dosing regimens or treatment duration on outcomes, which precludes definitive conclusions regarding whether the survival benefit reflects a class effect of prophylactic anticoagulation or is specific to standardized dosing protocols. Notably, our intentional exclusion of patients with conditions requiring therapeutic anticoagulation (including atrial fibrillation and VTE) aimed to isolate the effects of prophylactic enoxaparin use; however, this design choice may limit generalizability to broader populations of patients with COPD, as these comorbidities are prevalent in critically ill patients and may interact with thrombotic risks. Third, bleeding events, a critical safety endpoint, were not analyzed because of data limitations, hindering a comprehensive risk–benefit assessment, especially given the elevated bleeding susceptibility in patients with COPD due to factors, such as age-related frailty, renal impairment, and concomitant antiplatelet use. Finally, while an observational design allows for hypothesis generation, caUnited Stateslity has not been established. For example, clinicians may selectively prescribe enoxaparin to patients perceived as having a low baseline bleeding risk or a high likelihood of survival, thereby introducing potential confounding factors by indication. Prospective trials with protocolized dosing, rigorous bleeding surveillance, and serial biomarker monitoring are necessary to validate these findings and to refine anticoagulation strategies for critically ill patients with COPD.

## Conclusion

In this retrospective cohort study of critically ill patients with COPD, the initiation of prophylactic enoxaparin within 72 h of ICU admission was associated with a 48% decrease in ICU mortality. Enhanced benefits were observed in patients with high OASIS scores. These findings, bolstered by sensitivity analyses with an E-value of at least 3.26, suggest that early thromboprophylaxis may improve survival, particularly in patients with severe COPD in high-risk subgroups. While the results do not establish causality, they underscore the need for randomized controlled trials to confirm the efficacy of thromboprophylaxis, optimize dosing, and evaluate bleeding risk in this vulnerable population.

## Data Availability

The original contributions presented in the study are included in the article/supplementary material, further inquiries can be directed to the corresponding authors.
