# Correction: Association of early enoxaparin prophylactic anticoagulation with ICU mortality in critically ill patients with chronic obstructive pulmonary disease: a machine learning-based retrospective cohort study

**DOI:** 10.3389/fphar.2025.1636871

**Published:** 2025-06-10

**Authors:** Shan Lin, Jing Zhang, Xin Dang, Qingyuan Zhan

**Affiliations:** ^1^ Department of Respiratory and Critical Care Medicine, Affiliated Hospital of North Sichuan Medical College, Nanchong, Sichuan, China; ^2^ Department of Otolaryngology Head and Neck Surgery, Affiliated Hospital of North Sichuan Medical College, Nanchong, Sichuan, China; ^3^ Department of Pulmonary and Critical Care Medicine, Center of Respiratory Medicine, National Center for Respiratory Medicine, China-Japan Friendship Hospital, Beijing, China

**Keywords:** enoxaparin, prophylactic anticoagulation, chronic obstructive pulmonary disease, critical care, prognosis, machine learning

Jing Zhang was erroneously assigned as a corresponding author, and Qingyuan Zhan was erroneously omitted as a corresponding author. The correct corresponding authors are Shan Lin and Qingyuan Zhan.

Author Qingyuan Zhan was erroneously assigned as equal contributing author, and Jing Zhang was erroneously omitted as equal contributing author. The equal contribution statement applies to Shan Lin and Jing Zhang.

The original version of this article has been updated.

